# Variations in neurotoxicity and proteome profile of Malayan krait (*Bungarus candidus*) venoms

**DOI:** 10.1371/journal.pone.0227122

**Published:** 2019-12-30

**Authors:** Muhamad Rusdi Ahmad Rusmili, Iekhsan Othman, Syafiq Asnawi Zainal Abidin, Fathin Athirah Yusof, Kavi Ratanabanangkoon, Lawan Chanhome, Wayne C. Hodgson, Janeyuth Chaisakul

**Affiliations:** 1 Kulliyyah of Pharmacy, International Islamic University Malaysia, Kuantan Campus, Bandar Indera Mahkota, Kuantan, Pahang Darul Makmur, Malaysia; 2 Jeffrey Cheah School of Medicine and Health Sciences, Monash University Sunway Campus, Bandar Sunway, Malaysia; 3 Department of Microbiology, Faculty of Science, Mahidol University, Bangkok, Thailand; 4 Snake Farm, Queen Saovabha Memorial Institute, Thai Red Cross Society, Bangkok, Thailand; 5 Monash Venom Group, Department of Pharmacology, Biomedical Discovery Institute, Monash University, Clayton, VIC, Australia; 6 Department of Pharmacology, Phramongkutklao College of Medicine, Bangkok, Thailand; Instituto Butantan, BRAZIL

## Abstract

Malayan krait (*Bungarus candidus*) is a medically important snake species found in Southeast Asia. The neurotoxic effects of envenoming present as flaccid paralysis of skeletal muscles. It is unclear whether geographical variation in venom composition plays a significant role in the degree of clinical neurotoxicity. In this study, the effects of geographical variation on neurotoxicity and venom composition of *B*. *candidus* venoms from Indonesia, Malaysia and Thailand were examined. In the chick biventer cervicis nerve-muscle preparation, all venoms abolished indirect twitches and attenuated contractile responses to nicotinic receptor agonists, with venom from Indonesia displaying the most rapid neurotoxicity. A proteomic analysis indicated that three finger toxins (3FTx), phospholipase A_2_ (PLA_2_) and Kunitz-type serine protease inhibitors were common toxin groups in the venoms. In addition, venom from Thailand contained L-amino acid oxidase (LAAO), cysteine rich secretory protein (CRISP), thrombin-like enzyme (TLE) and snake venom metalloproteinase (SVMP). Short-chain post-synaptic neurotoxins were not detected in any of the venoms. The largest quantity of long-chain post-synaptic neurotoxins and non-conventional toxins was found in the venom from Thailand. Analysis of PLA_2_ activity did not show any correlation between the amount of PLA_2_ and the degree of neurotoxicity of the venoms. Our study shows that variation in venom composition is not limited to the degree of neurotoxicity. This investigation provides additional insights into the geographical differences in venom composition and provides information that could be used to improve the management of Malayan krait envenoming in Southeast Asia.

## Introduction

Snake envenoming is responsible for considerable mortality and morbidity worldwide. The highest burden of snakebite exists in tropical regions of Asia (*i*.*e*. South Asia and Southeast Asia), Papua New Guinea, African countries and Latin America [1]. Kraits (*Bungarus* sp.) are medically important snakes in Asia that are found throughout the Indian subcontinent, most parts of Southeast Asia and Southern China. The Malayan krait (*Bungarus candidus*) is found in Peninsular Malaysia, Indonesia (Sumatra, Java and Bali), Vietnam and Thailand. It is classified as a category 1 medically important venomous species in Indonesia and Thailand [2,3]. Interestingly, in Malaysia, *B*. *candidus* is only considered as a category 2 [4] species and envenoming is relatively rare [5].

The most significant effect of envenoming by *B*. *candidus* is progressive neuromuscular paralysis leading to respiratory failure. Cardiovascular disturbances (*i*.*e*. hypertension, tachycardia and shock) [6], myotoxicity, hyponatraemia and rhabdomyolysis have also been reported [7]. In addition, myotoxicity and nephrotoxicity were recently reported in experimentally envenomed animals [8], which corelated to previous clinical reports from Vietnam [7]. Other anomalies such as brain damage due to anoxia, cerebral ataxia, and mydriasis have also been observed in envenomed patients [9].

Early antivenom administration and respiratory support are essential for management of systemic *B*. *candidus* envenoming. The Queen Saovabha Memorial Institute (Thai Red Cross Society, Bangkok, Thailand) is the sole manufacturer of *B*. *candidus* antivenom (BCAV). They also produce Neuro Polyvalent Snake antivenom (NPAV) for Southeast Asian elapid envenoming which covers the venoms of *Ophiophagus hannah*, *Naja kaouthia*, *B*. *fasciatus* and *B*. *candidus* [10]. It has been reported that BCAV minimizes hospitalization time for *B*. *candidus* bite victims in Thailand [11]. Although *B*. *fasciatus* monovalent antivenom (BFAV) has been shown to have neutralizing effects against three specific kraits found in Thailand [12], neither BFAV nor BCAV cross neutralized the *in-vitro* skeletal muscle effects of venoms from other *Bungarus* species [13]. In addition, administration of antivenom at a higher concentration than recommended was required to prevent *in-vitro* neurotoxic activity [13].

Neurotoxicity observed following envenoming by kraits is attributed to the presence of two major types of neurotoxins *i*.*e*. pre- and post-synaptic neurotoxins [14,15]. Pre-synaptic neurotoxins interrupt neurotransmitter release, synthesis, storage or turnover in the synaptic nerve terminal [16], while post-synaptic neurotoxins inhibit the interaction of ACh with the skeletal muscle nicotinic receptor. Proteome analysis of Malaysian *B*. *candidus* venom found that PLA_2_, three-finger toxins (3FTxs) and Kunitz-type inhibitors are the major components [17]. In addition, high molecular weight enzymes *i*.*e*. L-amino acid oxidase, hyaluronidase including some unique proteins such as natriuretic peptide, vespryn and serine protease families were detected [17].

Geographical variation in venom composition has been shown to have a significant effect on antivenom efficacy [18–20]. Previous pharmacological, biochemical and proteomic analyses of several venomous snake species have reported differences in biological activities and composition of venom from the same snake species from different geographical localities [21–23]. Even though *B*. *candidus* envenoming is significant in many regions of Southeast Asia, studies regarding geographical variation of *B*. *candidus* venom composition are limited. In this study, we examined potential variations in the venom proteomic and pharmacological activity of venoms from *B*. *candidus* specimens collected from three different geographical localities i.e. Indonesia, Malaysia and Thailand. The efficacy of BCAV from QSMI against the *in-vitro* neurotoxicity caused by these venoms was also evaluated.

## Material and methods

### Animal ethics and care

Male Leghorn chicks (*Gallus gallus domesticus*) (4–10 days old) were purchased from a local poultry hatchery (Bangkok, Thailand) and kept in a well-lit cage with access to food and drinking water *ad libitum*. Approvals for all experimental procedures were granted from the Subcommittee for Multidisciplinary Laboratory and Animal Usage of Phramongkutklao College of Medicine (Documentary Proof of Ethical Clearance no: IRBRTA 222/2562) in accordance with the U.K. Animal (Scientific Procedure) Act, 1986 and the National Institutes of Health guide for the care and use of Laboratory animals (NIH Publications No. 8023, revised 1978).

### Venom preparation and storage

Indonesian *B*. *candidus* venom (BC-I) was a gift from PT BioFarma Bandung, Indonesia. The venom was milked from several specimens caught in West Java, Indonesia. Malaysian *B*. *candidus* venom (BC-M) was milked from 10 specimens captured in Northwest Peninsular Malaysia. The specimens were milked 3 times with interval of 3 weeks between milking before being released at the area of capture. The research permit for Malaysian *B*. *candidus* was provided by the Department of Wildlife and National Parks, Government of Malaysia (Permit no.: HQ-0067-15-70). *B*. *candidus* Thailand (BC-T) venom was purchased from Snake Farm of Queen Saovabha Memorial Institute (QSMI) of the Thai Red Cross Society, Bangkok. The venoms were extracted from 3 specimens captured in Nakhon Si Thammarat, Southern Thailand. *B*. *candidus* venom from each locality was pooled before being frozen and freeze-dried. Freeze-dried venom samples were weighed, labeled and stored at -20°C prior to use. When required, the venoms were weighed and dissolved in distilled water. Dissolved venoms were kept on ice during experiments.

### Protein concentration

Venom protein was determined using a BCA Protein Assay Kit (Pierce Biotechnology; Illinois, USA) as per manufacturer’s instructions. In brief, 25 μL of venom was loaded onto a 96-well plate in triplicate. Then 200 μL of reagent buffer mix was added to each well. The plate was incubated at 37°C for 30 min, then read at 562 nm using an ELISA plate reader spectrophotometer (Enspire^®^ multimode plate reader, Waltham, MA, USA). Protein concentration of the venom was determined from the standard curve.

### Sodium dodecyl sulphate-polyacrylamide gel electrophoresis (SDS–PAGE)

Venoms (10 μg) in reducing and non-reducing sample buffers were resolved and electrophoresed at 90 V in 12% separating gel with 5% stacking gel using the method previously described [24]. Protein bands were visualized by staining with X-Press Blue Protein Stain (Himedia, LBS. Marg, Mumbai, India), followed by de-staining using distilled water. TriColor Broad Protein Ladder (Biotechrabbit GmbH, Henigsdorf, Germany) was electrophoresed in the gel as protein molecular weight marker. The gel was scanned using Chemi Imager, Alliance Mini HD9 Auto (UVITEC, Cambridge UK) and analyzed using ImageJ software [25].

### Western blot

Venoms (10 μg) were resolved on a 12% SDS-PAGE gel and transferred onto a PVDF membrane (Merck Millipore, Billerica, MA, USA) using wet electroblotting (Cleaver Scientific, Warwickshire, UK) at 300 mA for 45 min. The membrane was then blocked in 5% skim milk in TBST (20 mM Tris, 0.5 M NaCl, 0.5% Tween-20) to prevent non-specific binding and then incubated with primary antibody (BCAV diluted 1:500-fold in TBST with 5% skim milk) overnight at 4˚C. The membrane was then washed three times for 30 min with TBST buffer. Immunoreactive bands were visualised using appropriate secondary antibodies (goat-anti-horse-IgG-HRP, Santa Cruz Biotechnology, Dallas, TX, USA) and western chemiluminescence ECL detection reagent (Cyanagen Srl; Bologna, Italy). The membrane was scanned using Chemi Imager, Alliance Mini HD9 Auto (UVITEC, Cambridge UK).

### Reverse-phase HPLC

Venoms (100 μg) were dissolved in Milli-Q grade water at a final concentration of 1 mg/ml before being centrifuged at 10,000 rpm for 5 min. The supernatants (20 μL) were loaded into a Jupiter 5 μm C18 300 Å reverse phase column (Phenomenex, Torrance, CA, USA) mounted on an Agilent 1260 Infinity high pressure liquid chromatography system (Agilent Technologies, Santa Clara, CA, USA). The column was equilibrated with 0.1% trifluroacetic acid in water (solution A) and the peaks were eluted from the column with 90% acetonitrile in 0.1% trifluroacetic acid in water (solution B) using the following gradient; 15% solution B from 0–10 min, 15–80% solution B from 10–70 min and 80–100% solution B from 70–80 min at flow rate of 1 ml/min. The eluted peaks were monitored at 214 nm using ChemStation software (Agilent Technologies, Santa Clara, CA, USA). Fractions corresponding to peak elution were manually collected.

### In-solution digestion of collected fractions

Ammonium bicarbonate (25 μL of 100 mM), trifluroethanol (25 μL) and DTT (1 μL of 200 mM) were added into vials containing freeze-dried fractions. The mix was then briefly vortexed, centrifuged and incubated at 60°C for 1 h. Iodoacetamide (4 μL of 200 mM) was added into the tubes and left for 1 h in the dark. Then, 1 μL of DTT was added into the tubes and left for 1 h at room temperature. The sample pH was adjusted to 7–9 using Milli-Q water and 100 mM ammonium bicarbonate before trypsin addition. The vials were then incubated overnight at 37°C. The trysin reaction was stopped at the end of the incubation using 1 μL of formic acid. The samples were dried using a vacuum concentrator and stored at -20°C prior to analysis. The sample was re-dissolved by adding 10 μL of 0.1% formic acid into each sample tube before being vortexed and centrifuged prior to loading into an ESI-LCMS/MS system.

### Nanoflow liquid chromatography-ionization coupled with mass spectrometry/mass spectrometry (ESI-LCMS/MS)

Digested sample (1 μL) was loaded into an Agilent C18 300 Å Large Capacity Chip (Agilent Technologies, Santa Clara, USA) mounted on an Agilent 1200 HPLC-Chip/MS Interface, coupled with Agilent 6550 iFunnel Q-ToF LC/MS (Agilent Technologies, Santa Clara, USA). The flow rate was set at 4 μL/min for the capillary pump and 0.5 μL/min for the nano pump. The column was equilibrated with 0.1% formic acid in water (solution A) and digested peptides were eluted with an increasing gradient of 90% ACN in 0.1% formic acid using the following gradient; 0–75% from 0 to 30 min and 75% for 4 min. The mass spectrometry was set at positive ion polarity mode. The capillary voltage was set at 2050 V and the fragmentor voltage was set at 360 V. The drying gas flow was set at 5 L/min and gas temperature at 325°C.

### Main venom protein identification

Venom proteins were identified using PEAK Studio (version 7.0, Bioinformatics Solution, Waterloo, Canada). The homology search was conducted by comparing de novo sequence tag with UniProt Serpentes database from July 2017. Carbamidomethylation was set as the fixed modification and trypsin as the digestion enzyme. Parent mass error tolerance and fragment mass error tolerance were set at 0.1 Da. Protein was accepted if they fulfilled the following criteria; the maximum number of missed cleavages and maximum variable pot-translational modification per peptide is 3, false detection rate (FDR) is less than 0.1%, the minimum value for protein -10logP is 30 and the minimum number of unique peptides is 2.

### Chick biventer cervicis nerve-muscle preparation

Male chicks (4–10 days old) were killed by asphyxiation using CO_2_ and the biventer cervicis nerve-muscles removed. The tissues were mounted in 5 ml organ baths containing physiological salt solution (118.4 mM NaCl, 4.7 mM KCl, 1.2 mM MgSO_4_, 1.2 mM KH_2_PO_4_, 2.5 mM CaCl_2_, 25 mM NaHCO_3_ and 11.1 mM glucose). The solution was maintained at 34°C and bubbled with carbogen (95% O_2_ and 5% CO_2_) under 1 g resting tension. The tissues were indirectly stimulated every 10 s for a duration of 0.2 ms at supramaximal voltage using a Grass SD9 stimulator. *d*-Tubocurarine (dTC; 10 μM) was added to the organ bath when muscle twitches were consistent, and the subsequent abolition of twitches confirmed the selective stimulation of the motor nerve. Responses to nerve stimulation were re-established by thorough washing. Contractile responses to the nicotinic receptor agonists, acetylcholine (ACh; 1 mM for 30 s) and carbachol (CCh; 20 μM for 60 s), and a membrane depolarizing agent, potassium chloride (KCl; 40 mM for 30 s) were obtained in the absence of electrical stimulation. The preparations were then equilibrated for at least 30 min with continuous nerve stimulation (as described above) before addition of venom. In all experiments, venom (3–10 μg/ml) was left in contact with the preparation until responses to nerve stimulation were abolished or up to 4 h if total twitch blockade did not occur. Twitch responses were measured following the addition of venom using Grass force displacement transducers (FT03) and recorded using a MacLab System. Time taken to reduce the amplitude of the indirect twitches by 90% (*t*_*90*_) was used as a quantitative measure of *in-vitro* neurotoxicity.

### Determination of PLA_2_ Activity

PLA_2_ activity for each *B*. *candidus* venom was determined using a secretory PLA_2_ colourmetric assay kit (Cayman Chemical, USA) according to manufacturer’s instructions. In brief, the 1, 2-dithio analog of diheptanoyl phosphatidylcholine was used as a substrate for venom PLA_2_ enzymes. Free thiols generated following the hydrolysis of the thio ester bond at the *sn*-2 position by PLA_2_ are detected using DTNB (5, 5’-dithio-bis-(2-nitrobenzoic acid)). The change of absorbance was monitored at 405 nm using a plate reader spectrophotometer (EnSpire® Multimode Plate Reader, Perkin Elmer, USA). The absorbance was sampled every minute for 10 min period. PLA_2_ activity was expressed as micromoles of phosphatidylcholine hydrolyzed per minute per milligram of enzyme. Three separate determinations of PLA_2_ activity were done in triplicate for all samples including positive control, bee venom (*n* = 3).

### Chemicals and drugs

Monovalent *B*. *candidus* antivenom (BCAV; Lot No.: BC00115; Expiry date: 30-1-2020) was purchased from Queen Saovabha Memorial Institute (QSMI) of the Thai Red Cross Society, Bangkok, Thailand. The following drugs and consumables were purchased from Sigma Aldrich (St. Louis, MO, USA): ACh, CCh, *d*-tubocurarine, formic acid, NaCl, KCl, MgSO_4_, KH_2_PO_4_, CaCl_2_, NaHCO_3_ and glucose. HPLC-grade and LCMS-grade acetonitrile were purchased from Fisher Scientific (Loughborough, Leicestershire, UK).

### Data analysis and statistics

Statistical analysis was performed using Prism 6.0 software (GraphPad Software, La Jolla, CA, USA). Twitch height and contractile responses to agonists were expressed as a percentage of the corresponding value prior to the administration of venoms. Multiple comparisons were made using a one-way analysis of variance (ANOVA) followed by a Bonferroni multiple comparison test. Values of *P* < 0.05 were accepted as significant. Data were expressed as mean ± SEM.

## Results

### Sodium dodecyl sulphate-polyacrylamide gel electrophoresis (SDS–PAGE) and Western blot

**Fig 1:**
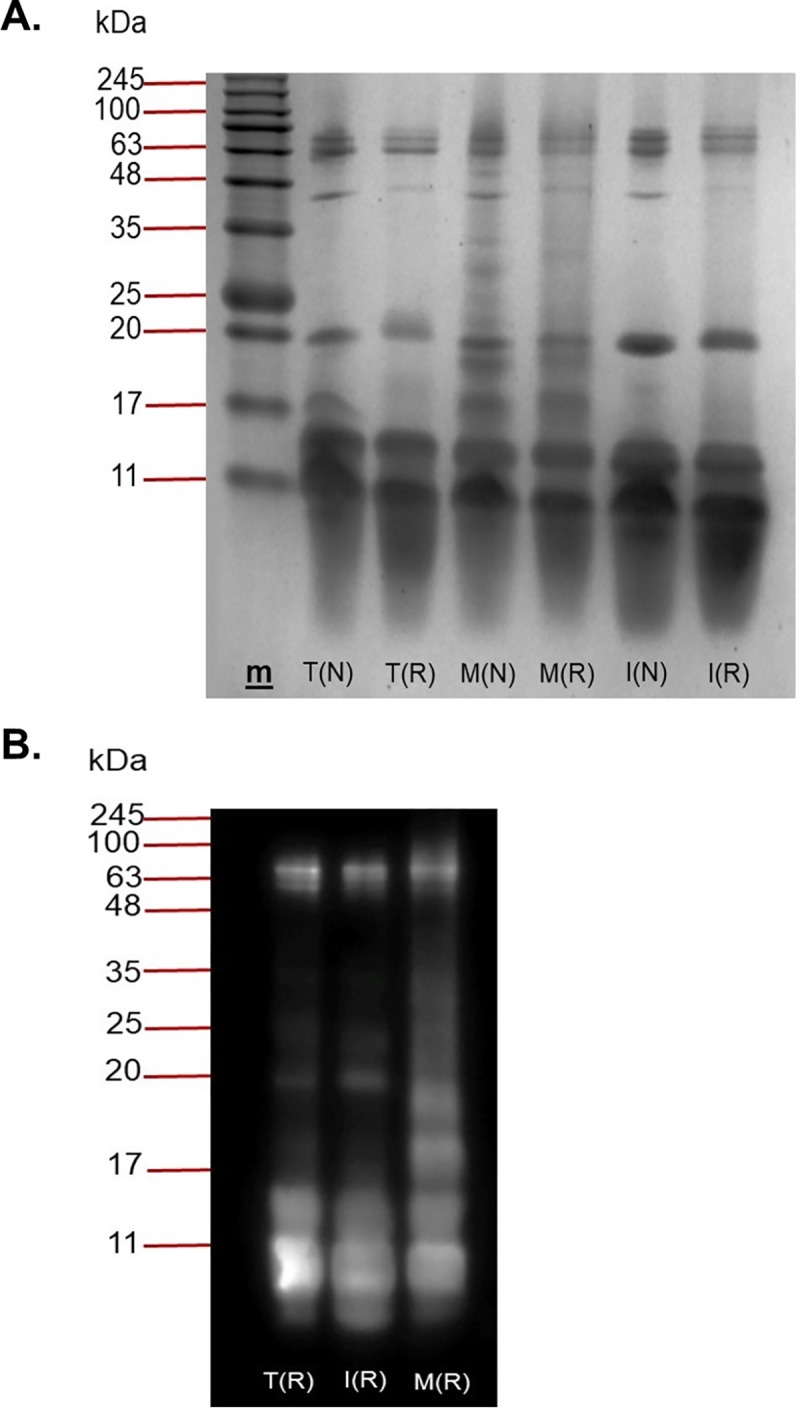
(A) SDS-PAGE and (B) Western immunoblotting of *B*. *candidus* venoms on a 12% separating gel with 5% stacking gel. Venoms were treated in reducing (R) or non-reducing buffer (N) prior to loading, electrophoresis, and stained with Coomassie Blue. T indicates *B*. *candidus* venom from Thailand, M indicates *B*. *candidus* venom from Peninsular Malaysia and I indicates *B*. *candidus* venom from Indonesia. (R) indicates venom treated with reducing sample buffer and (N) indicates venom treated with non-reducing sample buffer. Western immunoblotting of reduced *B*. *candidus* venoms incubated with monovalent *B*. *candidus* antivenom. **m** is molecular weight marker.

**Fig 2:**
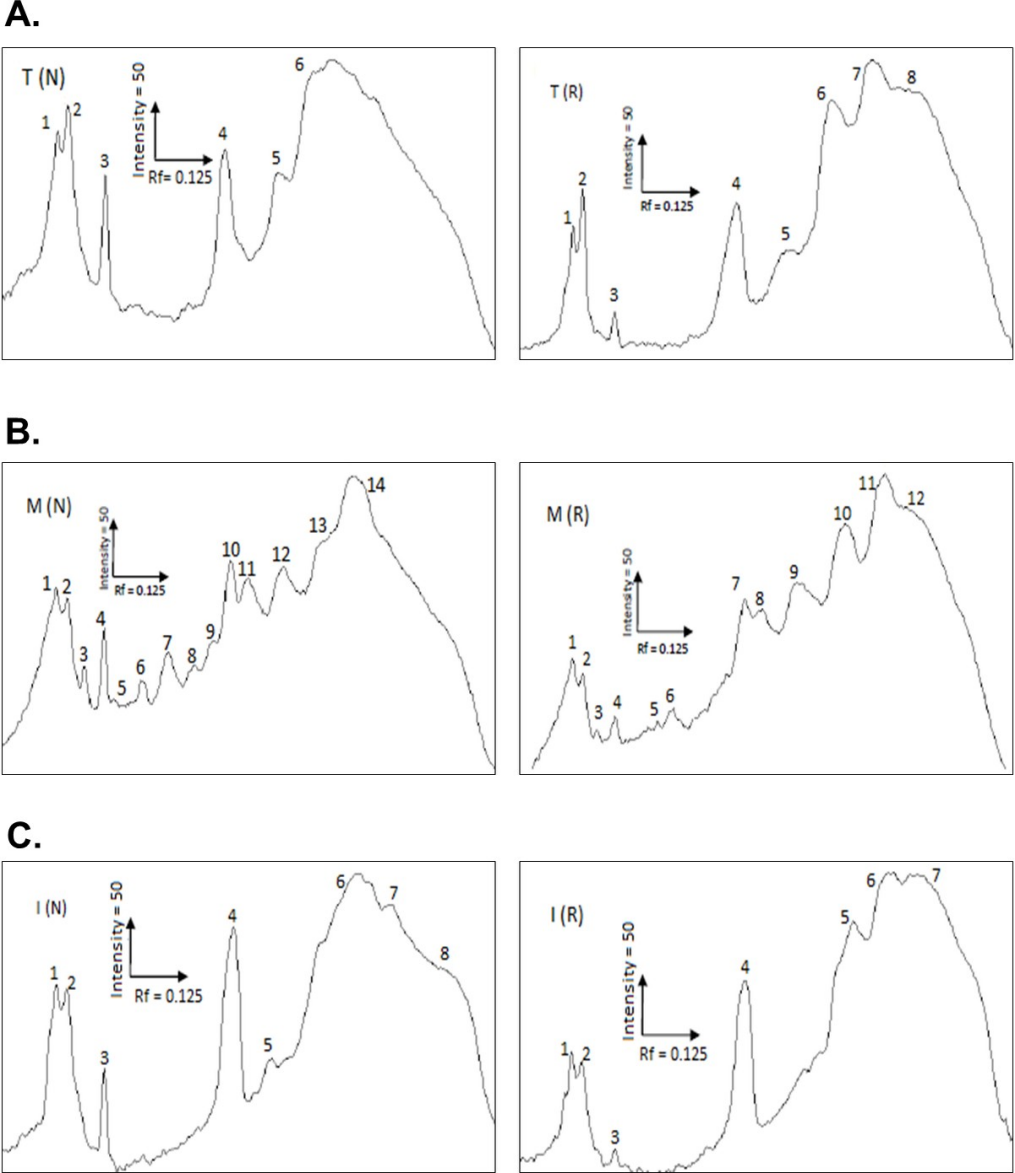
Densitogram for loaded lanes in SDS-PAGE:(A) Lanes loaded with BC-T, (B) Lanes loaded with BC-M and (C) Lanes loaded with BC-I.

*B*. *candidus* venoms from Thailand (BC-T), Malaysia (BC-M) and Indonesia (BC-I) were resolved in a gel under reducing and non-reducing conditions (Fig 1A). SDS-PAGE analysis of venoms shows that there were differences in intensity and pattern of protein bands (Fig 1A). BC-M venom possessed a greater number of protein bands compared to BC-I and BC-T venom. Thick and high intensity bands were observed in the MW range below 17 kDa in reduced and non-reduced BC venom. No protein bands were observed within the range of 25–35 kDa in reduced and non-reduced BC-I and BC-T venoms (Fig 1A). Densitogram for the lanes loaded with BC-M (Fig 2B) showed that 12 peaks were detected in reduced sample whereas 14 peaks were detected in non-reduced sample. In reduced BC-T (Fig 2A), 8 peaks were detected and 7 peaks in BC-I (Fig 2C). The number of peaks detected in non-reduced BC-T and BC-I is 6 and 8, respectively (Fig 2A and 2C). Western blot analysis showed that BCAV was able to detect most proteins in venoms from all localities (Fig 1B).

### Effect of venoms on the chick biventer cervicis nerve-muscle preparation

**Fig 3:**
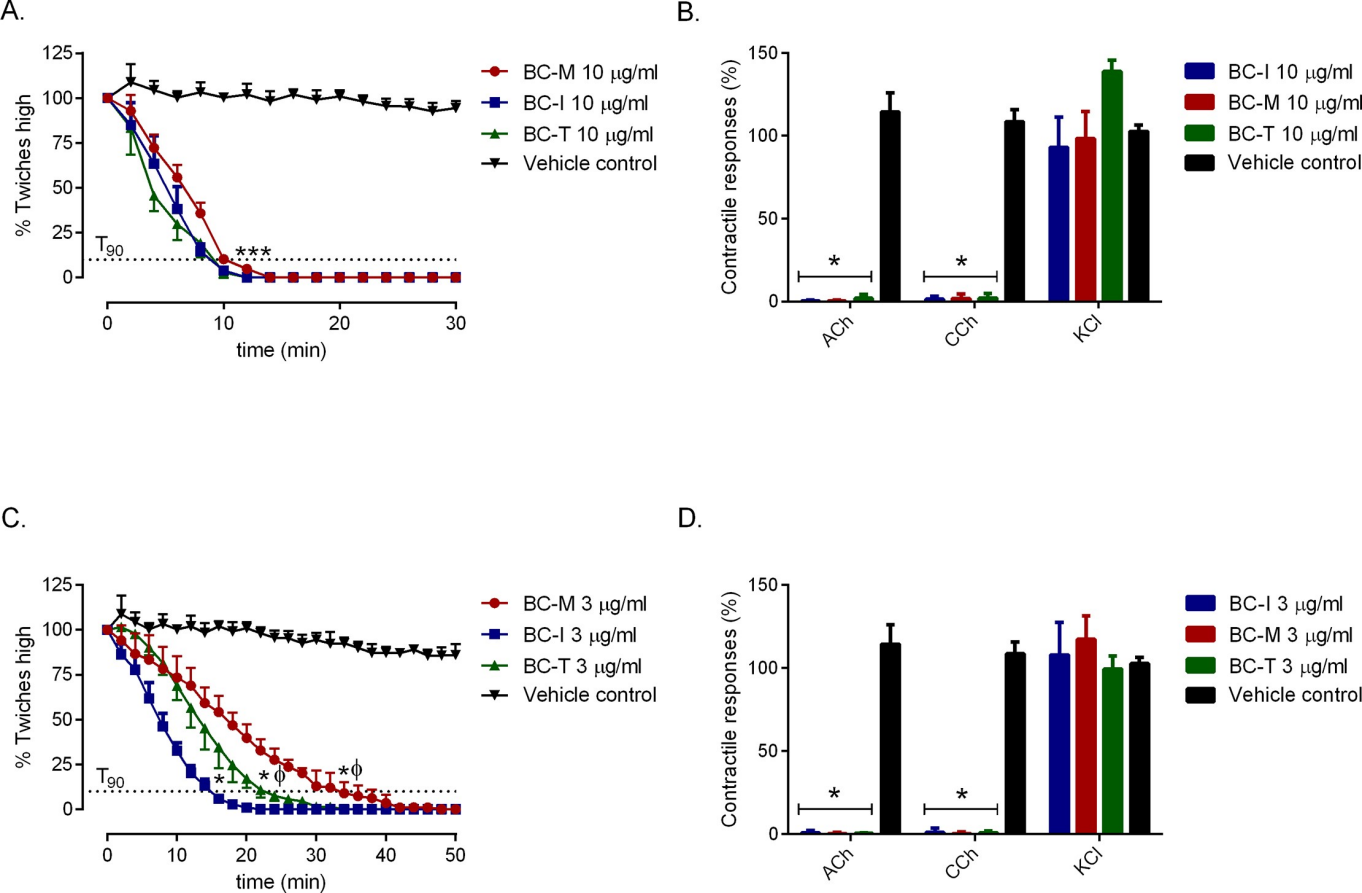
Effect of *B*. *candidus* venoms at 10 μg/ml (A.) and 3 μg/ml (C.) on indirect twitches of the chick biventer cervicis nerve-muscle preparation including responses to exogenous agonists (B and D). * significantly different from vehicle control; *** significantly different from vehicle control in twitch height of all three *B*. *candidus* venoms; ϕ significantly different from BC-I (*n* = 4, one-way ANOVA, *P* < 0.05).

*B*. *candidus* venoms (3 and 10 μg/ml) from all localities caused a significant reduction in twitch height compared to vehicle (i.e. BSA) (Fig 3A and 3C: *n* = 4). At 10 μg/ml (Fig 3A), geographical variants did not show significant difference in the time required for the twitches to be reduced by 90% (i.e. *t*_*90*_ ~ 10 min, Table 1). At a concentration of 3 μg/ml (Fig 3C), the effects of BC-M venom were significantly slower (i.e. *t*_90_ = 36.0 ± 4.1 min) compared to BC-T (*t*_90_ = 22.0 ± 1.6 min) and BC-I (*t*_*90*_ = 14.2 ± 0.5 min). All three venoms at 3 and 10 μg/ml abolished contractile responses to exogenous ACh (1 mM) and CCh (20 μM), but had no significant effect on responses to KCl (40 mM) (Fig 3B and 3D). Vehicle had no significant inhibitory effect on the contractile responses to exogenous agonists (*n* = 4; one-way ANOVA, *P* < 0.05).

**Table 1:** Comparison of elapid venom PLA_2_ activity and the time taken to cause 90% inhibition of nerve-mediated twitches (*t*_90_ values); N/A: Not available.

venom	PLA_2_ activity (μmol/min/mg)	*t*_90_ at 3 μg/ml (min)	*t*_90_ at 10 μg/ml (min)
*B*. *candidus* venom: Indonesia (BC-I)	3041 ± 128 (*n* = 3)	14.2 ± 0.5 (*n* = 4)	8.8 ± 0.5 (*n* = 4)
*B*. *candidus* venom: Malaysia (BC-M)	3225 ± 233 (*n* = 3)	36.0 ± 4.1 (*n* = 4)	10.3 ± 0.5 (*n* = 4)
*B*. *candidus* venom: Thailand (BC-T)	5694 ± 815 (*n* = 3)	22.0 ± 1.6 (*n* = 4)	9.0 ± 1.4 (*n* = 4)
*B*. *fasciatus* venom: Malaysia	77.2 ± 4.9 (*n* = 3) [14]	N/A	22.5 ± 5.0 (*n* = 3–4)[13]
*O*. *scutellatus* venom: Australia	N/A	95.7 ± 8.7 (*n* = 4)[29]	63.5 ± 5.7 (*n* = 4)[29]
*O*. *scutellatus* venom: Papua New Guinea	373.0 ± 32.6 (*n* = 3) [33]	N/A	44.0 ± 5.0 (*n* = 4)[28]
*P*. *textilis* venom: Australia	N/A	24.1 ± 1.7 (*n* = 4)[29]	10.7 ± 1.1(*n* = 4)[29]
Bee venom	536 ± 16 (*n* = 3)	N/A	N/A

### Antivenom studies

**Fig 4:**
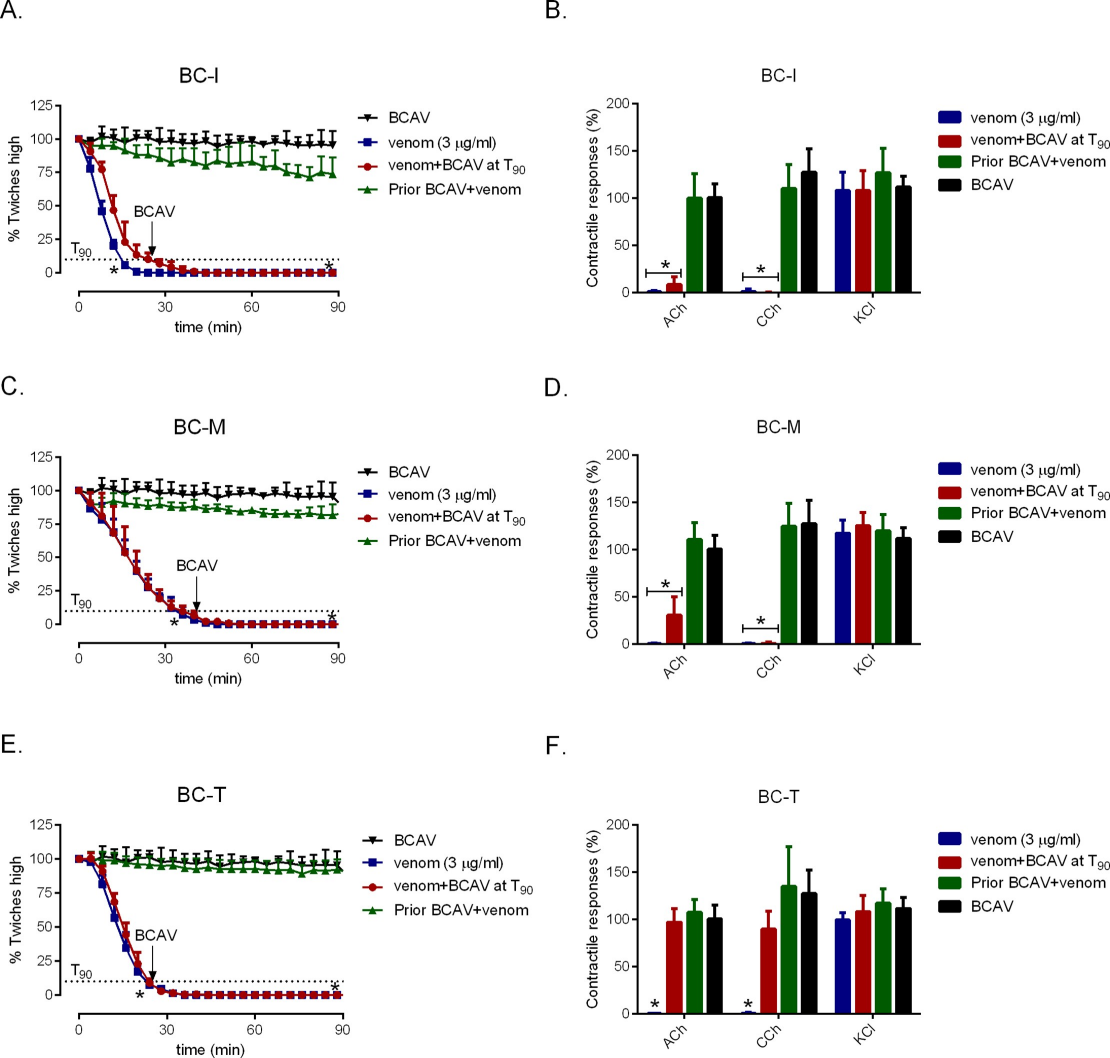
Effect of prior addition of *B*. *candidus* antivenom (BCAV; 1x the recommended titre) on indirect twitches in the presence of BC-I (A), BC-M (C) and BC-T (E). The contractile responses to exogenous agonists (i.e. ACh, CCh and KCl) of the chick biventer cervicis nerve-muscle preparation in the presence of BC-I (B), BC-M (D) and BC-T (F). * significantly different from *B*. *candidus* antivenom alone (*n* = 4, one-way ANOVA, *P* < 0.05).

Pre-incubation of BCAV at 1x the recommended titer (1 mL per 0.4 mg of *B*. *candidus* venom) for 10 min prior to the addition of *B*. *candidus* venoms (3 μg/ml) significantly delayed inhibition of twitch height in the chick biventer (Fig 4A, 4C and 4E: *n* = 4; one-way ANOVA, *P* < 0.05) and also prevented the inhibitory effect of venoms on contractile responses to exogenous nicotinic receptor agonists (Fig 4B, 4D and 4F). However, BCAV (3x recommended titer) did not reverse twitch inhibition when added at the *t*_*90*_ time point (Fig 4A, 4C and 4E). Interestingly, addition of BCAV at *t*_*90*_ in tissue that was exposed to BC-T (3 μg/ml) restored the contractile responses to ACh and CCh (Fig 4F; *n* = 4; one-way ANOVA, *P* < 0.05).

### Reverse-phase high performance liquid chromatography (RP-HPLC)

**Fig 5:**
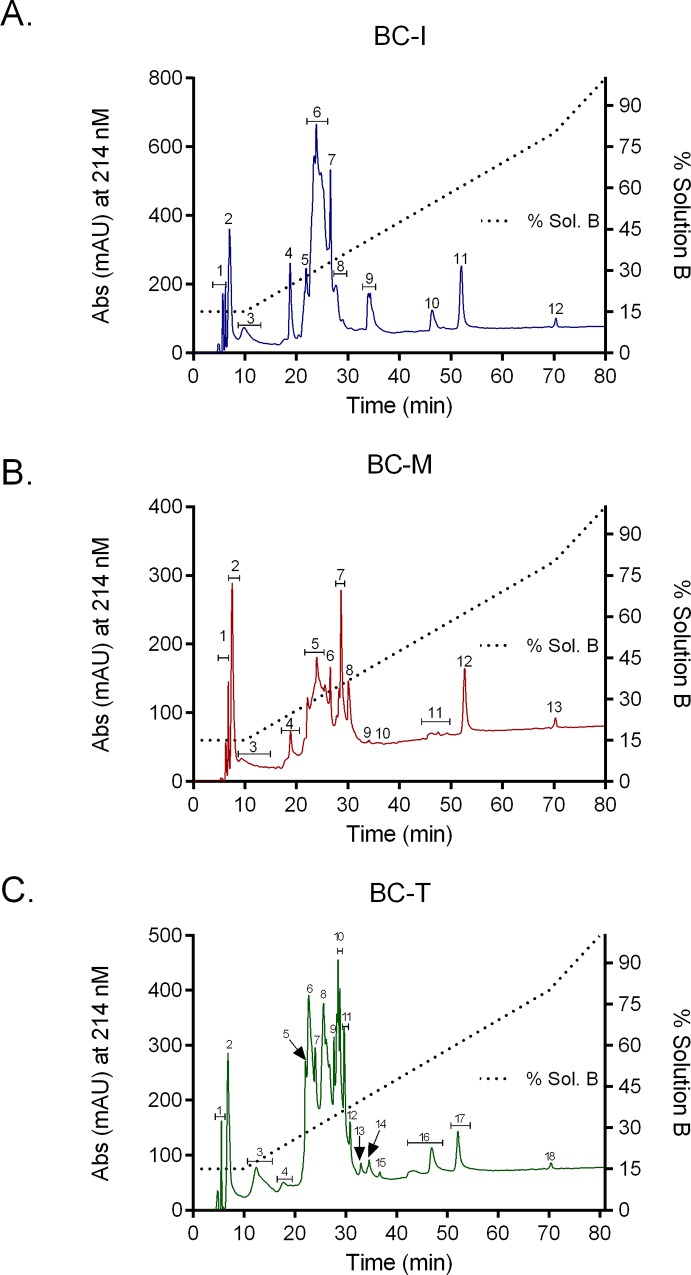
RP-HPLC chromatogram of (A) *B*. *candidus* venom from Bandung, Indonesia; BC-I (B), *B*. *candidus* venom from Peninsular Malaysia; BC-M and (C) *B*. *candidus* venom from Nakhon Si Thammarat, Southern Thailand; BC-T: run with the same conditions on a Jupiter analytical column, equilibrated with 0.1% trifluoroacetic acid in water (solution A) and eluted with solvent B (90% acetonitrile in 0.1% trifluoroacetic acid in water) using the following gradient; 15% solution B from 0–10 min, 15–80% solution B from 10–70 min and 80–100% solution B from 70–80 min at flow rate of 1 ml/min.

*B*. *candidus* venoms were profiled using RP-HPLC to determine differences in venom composition. Marked differences in chromatogram of venom profiles (Fig 5A–5C) were detected as the followings; 12 peaks were eluted for BC-I (Fig 5A), 13 peaks for BC-M (Fig 5B) and 18 peaks for BC-T venom (Fig 5C).

### Identification of main venom proteins

**Fig 6:**
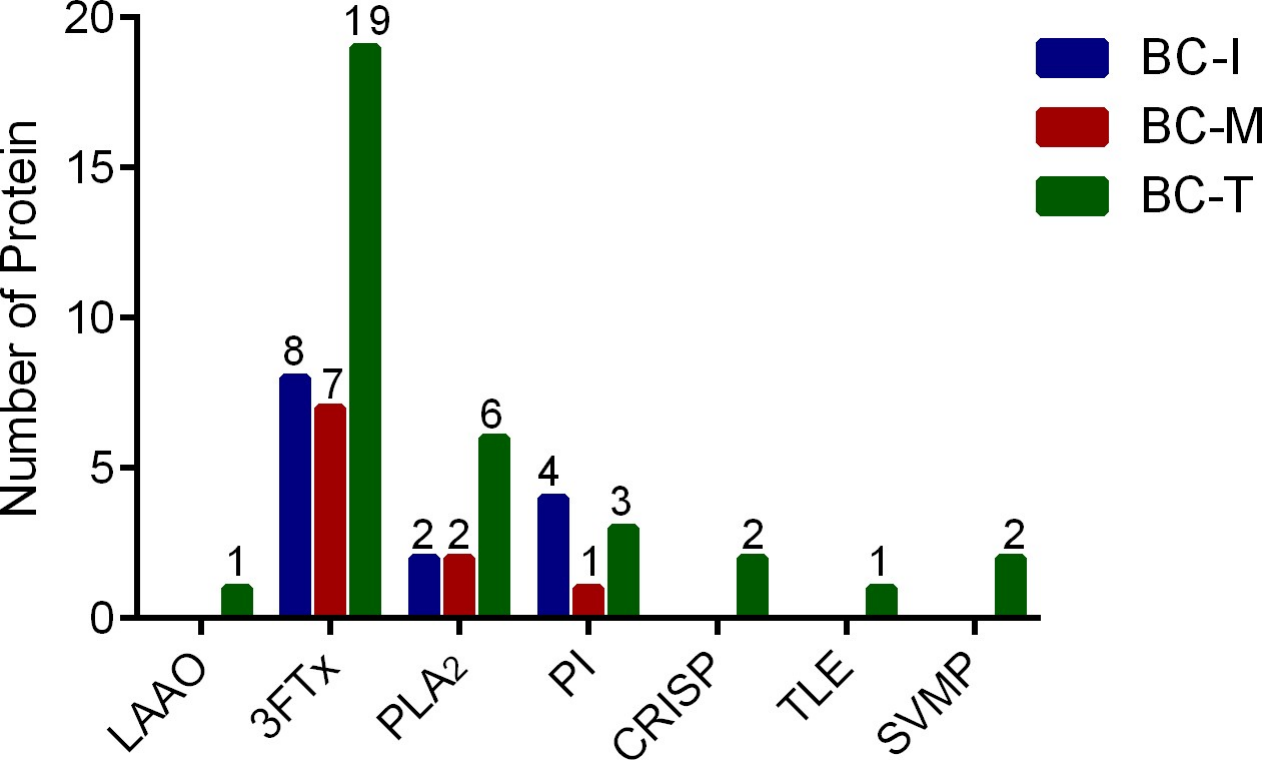
Number and group of venom proteins detected in Malayan krait from 3 different localities. 3FTx: three finger toxins, PLA_2_: phospholipase A_2_, LAAO: L-amino acid oxidase, CRISP: cysteine rich secretory protein, TLE: thrombin-like enzyme, SVMP: snake venom metalloproteinase, PI: Kunitz-type serine protease inhibitors.

**Fig 7:**
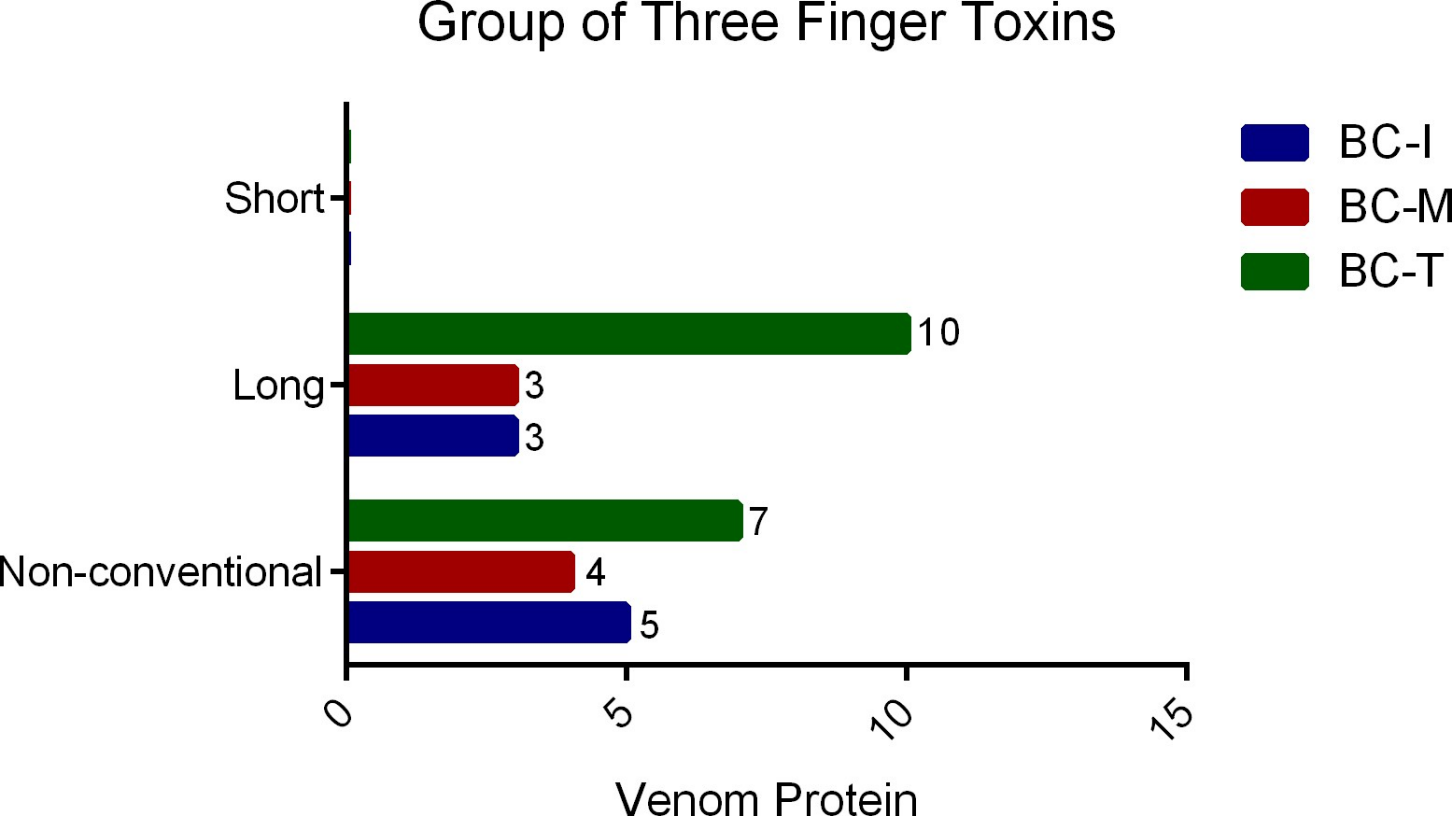
Three finger toxins detected in venom from three different localities.

Thirty-three proteins were detected in BC-T venom (S3 Table) whereas 14 proteins were detected in BC-I venom (S2 Table) and 9 proteins in BC-M venom (S1 Table). Three groups of proteins were detected in all 3 geographical variants, namely; three finger toxins (3FTx), PLA_2_s and Kunitz-type serine protease inhibitors (PI) (Fig 6). In addition to these 3 groups, L-amino acid oxidase (LAAO), cysteine rich secretory protein (CRISP) and snake venom metalloproteinase (SVMP) were also detected in BC-T venom (Fig 6). Interestingly, thrombin-like enzyme (TLE) was only detected in BC-T venom. 3FTx’s were the main venom protein group in all 3 venoms (Fig 6). Close examination of the 3FTx group showed that short-chain neurotoxins were not detected in the venoms (S1 Table, S2 Table, S3 Table and Fig 7). BC-T venom contained the highest number of detected long-chain neurotoxins and non-conventional toxins compared to BC-I and BC-M venoms (Fig 7). Alpha-bungarotoxins and beta-bungarotoxin subunits were detected in all 3 venoms (S1 Table, S2 Table, S3 Table).

### PLA_2_ activity

BC-T was found to have the highest PLA_2_ activity (5694 ± 815 μmol/min/mg; *n* = 3, Table 1). Whereas, PLA_2_ activity for BC-I and BC-M venoms was 3041 ± 128 and 3226 ± 233 μmol/min/mg (*n* = 3), respectively. The PLA_2_ activity for the positive control, i.e. bee venom, was 536 ± 16 μmol/min/mg (*n* = 3, Table 1).

## Discussion

*Bungarus candidus* is an endemic krait species in South East Asia. Severe neurotoxic and non-neurotoxic effects are observed following envenoming by *B*. *candidus* in Indonesia and Thailand [3,26]. However, Malaysian *B*. *candidus* envenoming is not known to cause significant non-neurotoxic effects [27]. This is partly because *B*. *candidus* envenoming is relatively uncommon in Malaysia compared to Indonesia and Thailand [5]. In the present study, we have demonstrated geographical variation in the composition and neurotoxicity of *B*. *candidus* venoms from 3 different localities.

Neurotoxic symptoms *i*.*e*. bilateral ptosis, persistently dilated pupil, limb weakness, breathlessness, hypersalivation, dysphonia and dysphagia are clinically important in the diagnosis and management of *B*. *candidus* envenoming [7]. Our data demonstrated that all venoms abolished contractile responses to acetylcholine and carbachol but not KCl (Fig 3B and Fig 3D). This indicates the presence of post-synaptic neurotoxins and a lack of myotoxicity in the venoms. Due to the complex regulatory requirements to gain approval for murine LD_50_ evaluations in many countries, determination of *t*_90_ values from the *in-vitro* isolated skeletal muscle preparations is used as an alternative. Based on *t*_*90*_ values, we observed the following order of potency in neurotoxic activity: BC-I>BC-T>BC-M (Table 1). Our results are in agreement with previous clinical work reporting the incidence and neurotoxic severity of *B*. *candidus* envenoming in the Southeast Asia [3,5,26].

In the present study, *B*. *candidus* venoms were found to be more potent compared to the venoms from known neurotoxic elapids previously characterized using the same chick-biventer cervicis nerve-muscle preparation in our laboratory (Table 1) [13,28,29]. However, there were no significant differences in neurotoxicity between *B*. *candidus* venoms at a concentration of 10 μg/ml but a significant difference was seen when a lower concentration (i.e. 3 μg/ml) was used. The neurotoxicity of whole venom does not solely depend on the toxicity of each neurotoxin but also on the quantity of each neurotoxin within the venom. Hence, at lower concentrations of whole venom, the quantity of each neurotoxin in the venom becomes more significant [23].

PLA_2_ toxins contribute to several pharmacological activities including neurotoxicity, myotoxicity, anticoagulation, smooth muscle relaxation/hypotension and hypersensitivity. However, the enzymatic activity of PLA_2_ is not completely related to its pharmacological activities [30,31]. Individual PLA_2_ enzyme display their own particular action [32]. PLA_2_ analysis indicates that BC-T venom contains by far the most PLA_2_ activity among tested venoms. It also has the highest number of PLA_2_ based on LC-MS results. Moreover, all *B*. *candidus* venoms also exhibited higher enzymatic activity compared to our previous data of *B*. *fasciatus* [14] and *O*. *scutellatus* [33] venoms. Our data also indicate that the degree of PLA_2_ activity in *B*. *candidus* venoms did not correlate with their order of neurotoxicity (Table 1).

Administration of BCAV or NPAV is the recommend treatment for systemic *B*. *candidus* envenoming. These antivenoms were found to be effective in reducing hospitalization and morbidity caused by *B*. *candidus* envenoming in Thailand [3,11]. NPAV was found to be effective in neutralizing Indonesian and Malaysian *B*. *candidus* when tested *in vivo* [10,34]. In addition, it has been shown that monovalent *B*. *fasciatus* antivenom is not effective in preventing *B*. *candidus*-induced *in vitro* neurotoxicity [13]. Unfortunately, there is no literature in the database on *in vivo* study of BCAV and its efficacy compared with NPAV. Geographical variation in venom composition is an important factor that affects the effectiveness [20] and quantity [35] of antivenom used in envenomed victims. In the present study, we have shown that prior incubation with BCAV, at the recommended titer, markedly delayed inhibition of indirect twitches produced by all *B*. *candidus* venoms. However, when BCAV at 3x recommended titer was added at the *t*_90_ time point, it failed to restore indirect twitches. The inability of antivenom to reverse neurotoxicity is in agreement with previous *in-vitro* studies [13], and indicates the likely presence of irreversible presynaptic neurotoxins in the venoms [36]. It is also possible that there are unique toxins in BC-M and BC-I which unable to be neutralized by the antivenom. In contrast, contractile responses of the chick biventer preparation to ACh and CCh were restored by the addition of antivenom at the *t*_90_ time point in the presence of BC-T venom. This phenomenon might be due to the high binding capacity of antivenom to the toxins, particularly the postsynaptic toxins in venom from Thailand which was used during the immunization for antivenom production.

Variation in animal venom composition can be classified as geographical, inter-and intra- species or even individual variation [37–39]. Proteomic techniques such as SDS-PAGE, liquid chromatography and mass spectrometry are commonly used to determine composition and variation of venom proteins [40–44]. Venom composition analysis using SDS-PAGE revealed a complex mixture of proteins with different molecular weights in *B*. *candidus* venoms. BC-M venom showed the greatest number of protein bands with highest intensity in the range between 48–17 kDa. It also has the highest number of peaks in its densitogram. When all venoms were reduced by 2-β mercaptoethanol, a higher number of protein bands within the molecular weight range 10–15 kDa were present. This is likely due to the reduction of multimeric PLA_2_ into individual units and the presence of three-finger toxins [45]. Although not many bands were detected in BC-I and BC-T-loaded lanes compared to BC-M in SDS-PAGE, nearly similar band patterns but with lower intensity were seen in Western blot. This indicates that most proteins in BC-M venom were also present in BC-I and BC-T venoms but in lower abundance. Reverse-phase HPLC chromatogram profile of the venoms showed variations in the number of peaks and peak intensity. This showed that the venom composition is unique for each locality. Although BC-M showed the most diverse pattern of protein bands using SDS-PAGE, the number of eluted peaks for BC-M was lower than BC-T. This is most likely due to the presence of similarly hydrophobic molecules but with different molecular weights in BC-M.

The 3FTx family was found to be the main venom protein component in the venoms from all three localities (Fig 6). The neurotoxic 3FTx can be divided into three subfamilies based on the number of amino acids in their primary sequence and the number of disulfide bonds, i.e. short-chain neurotoxins, long-chain neurotoxins, and non-conventional toxins [46–48]. A number of 3FTxs have been isolated from *B*. *candidus* venom i.e. bucandin [49], candoxin [50] and α-bungarotoxin [51]. α-Bungarotoxin is a long-chain 3FTx found in certain species of *Bungarus* [17,41,43,52]. In addition, αδ Bungarotoxins (αδ Bungarotoxin 1 and 2) were recently isolated from *B*. *candidus* venom from Thailand and shown to be more active at the interface of α-δ subunits of nicotinic acetylcholine receptors [53]. Venoms from all 3 localities showed the presence of multiple isomers of α-bungarotoxin, and short-chain neurotoxins were not detected in all samples (S1 Table, S2 Table, S3 Table). This finding is similar to earlier findings in Malaysian *B*. *candidus* [17]. Relatively rapid *in vitro* neurotoxicity of *B*. *candidus* venoms observed in the present study might be due to the inhibitory effect of postsynaptic neurotoxins on nicotinic acetylcholine in the chick biventer cervicis nerve-muscle preparation. The action of snake presynaptic neurotoxin appears to be slower than that of postsynaptic neurotoxins with a latency period up to 1 h [54]. Short-chain neurotoxins have been detected in two species of *Bungarus*, i.e. *B*. *fasciatus* [17] and *B*. *flaviceps* [43]. These two species lineages split earlier than other *Bungarus* species [55,56] and this could be the reason for the absence of short-chain neurotoxin in more advance *Bungarus* species which have more potent and irreversible neurotoxin components in their venoms [55].

In addition to the α-bungarotoxin, *Bungarus* species venoms are known to contain the presynaptic neurotoxin β-bungarotoxin, a type of PLA_2_ neurotoxin. This toxin consists of two protein subunits, i.e. chain A, which is a PLA_2,_ and chain B, a Kunitz-type protease inhibitor subunit. The presence of several Kunitz-types protease inhibitors and PLA_2_ chain A β-bungarotoxins indicates that β-bungarotoxins were present in all three samples. The highest number of PLA_2_ was detected in BC-T venom whereas the highest number of Kunitz-type protease inhibitors were detected in BC-I venom. This indicates that a higher number of β-bungarotoxin isoforms could be present in venom from Thailand and Indonesia compared to venom from Malaysia.

A thrombin-like enzyme that shared sequence similarity with Malayan pit viper thrombin-like enzyme was also detected in the Thailand venom. Similarly, previous work on Malaysian *B*. *candidus* venom did indicate the presence of several serine protease isoforms that showed sequence similarity with serine proteases and thrombin-like enzyme from various viperid species [17]. However, case reports of systemic envenoming by Thailand *B*. *candidus* did not indicate the occurrence of coagulopathy in envenomed victims [9,27]. More work will be needed to confirm the presence of this group of toxins in *B*. *candidus* venom and their role in envenoming. The number of detected venom protein and protein groups from all localities are lower than the number that was reported previously from Malaysian *B*. *candidus* [17]. Venom protein groups such as vespryn, trypsinogen, serine protease, nerve growth factor, hyalurodinase and acetylcholinesterase were not detected in the current study in venom from all localities. This is likely due to the variation in the amount of venom sample and equipment used in profiling and fraction collection.

In conclusion, our study confirms geographical variation in the composition of *B*. *candidus* venoms from 3 different localities. This variation may reflect differences of other geographical specific factors such as type of prey, ecology and climate. The knowledge regarding geographical variation of snake venom may provide additional insights into the clinical diagnosis and prediction of envenoming outcomes including a better therapeutic strategy and antivenom in the future.

## Supporting information

S1 Table List of proteins detected in Malaysian *B*. *candidus* venoms from an in-solution digests by LCMS/MS. (XLSX)

S2 Table List of proteins detected in Indonesian *B*. *candidus* venoms from an in-solution digests by LCMS/MS. (XLSX)

S3 Table List of proteins detected in Thai *B*. *candidus* venoms from an in-solution digests by LCMS/MS. (XLSX)

## Abbreviations

BFAV: *B*. *fasciatus* monovalent antivenom
BCAV: *B*. *candidus* monovalent antivenom
BC-T: *Bungarus candidus* from Southern Thailand
BC-I: *Bungarus candidus* from Indonesia
BC-M: *Bungarus candidus* from Peninsular Malaysia
Ach: Acetylcholine
CCh: carbachol
KCl: potassium chloride
3FTx: three finger toxins
PLA_2_: phospholipase A_2_
LAAO: L-amino acid oxidase
CRISP: cysteine rich secretory protein
TLE: thrombin-like enzyme
SVMP: snake venom metalloproteinase
HPLC: High Performance Liquid Chromatography
LCMS: Liquid Chromatography Mass Spectrometry

## References

[1] Gutierrez JM, Williams D, Fan HW, Warrell DA. Snakebite envenoming from a global perspective: Towards an integrated approach. Toxicon. 2010; 56(7): 1223–1235. 10.1016/j.toxicon.2009.11.020 19951718

[2] WHO. Guidelines for the Management of Snake-Bites. Guidelines for the Management of Snake-Bites. 2010

[3] Tongpoo A, Sriapha C, Pradoo A, Udomsubpayakul U, Srisuma S, Wananukul W, et al Krait envenomation in Thailand. Ther Clin Risk Manag. 2018; 14: 1711–1717. 10.2147/TCRM.S169581 30271155 PMC6145358

[4] WHO. Venomous snakes of the South-East Asia Region, their venoms and pathophysiology of human envenoming. Guidelines for the management of Snake-Bites, 2nd edition. 2016

[5] Chew KS, Khor HW, Ahmad R, Rahman NH. A five-year retrospective review of snakebite patients admitted to a tertiary university hospital in Malaysia. Int J Emerg Med. 2011; 4: 41 10.1186/1865-1380-4-41 21752254 PMC3143095

[6] Chaisakul J, Rusmili MR, Hodgson WC, Hatthachote P, Suwan K, Inchan A, et al A Pharmacological Examination of the Cardiovascular Effects of Malayan Krait (*Bungarus candidus*) Venoms. Toxins (Basel). 2017; 9. 3 29;9(4). pii: E122 10.3390/toxins9040122 28353659 PMC5408196

[7] Trinh KX, Khac QL, Trinh LX, Warrell DA. Hyponatraemia, rhabdomyolysis, alterations in blood pressure and persistent mydriasis in patients envenomed by Malayan kraits (*Bungarus candidus*) in southern Viet Nam. Toxicon. 2010; 56(6): 1070–1075. 10.1016/j.toxicon.2010.06.026 20637219

[8] Charoenpitakchai M, Wiwatwarayos K, Jaisupa N, Rusmili MRA, Mangmool S, Hodgson WC, et al Non-neurotoxic activity of Malayan krait (*Bungarus candidus*) venom from Thailand. J Venom Anim Toxins Incl Trop Dis. 2018; 24: 9 10.1186/s40409-018-0146-y 29556251 PMC5845229

[9] Laothong C, Sitprija V. Decreased parasympathetic activities in Malayan krait (*Bungarus candidus*) envenoming. Toxicon. 2001; 39: 1353–1357. 10.1016/s0041-0101(01)00087-3 11384723

[10] Leong PK, Sim SM, Fung SY, Sumana K, Sitprija V, Tan NH. Cross neutralization of Afro-Asian cobra and Asian krait venoms by a Thai polyvalent snake antivenom (Neuro Polyvalent Snake Antivenom). PLoS Negl Trop Dis. 2012; 6: e1672 10.1371/journal.pntd.0001672 22679522 PMC3367981

[11] Leeprasert W, Kaojarern S.Specific antivenom for *Bungarus candidus*. J Med Assoc Thai. 2007; 90: 1467–1476. 17710993

[12] Chanhome L, Wongtongkam N, Khow O, Pakmanee N, Omori-Satoh T, Sitprija V. Genus specific neutralization of *Bungarus* snake venoms by Thai Red Cross banded krait antivenom. J Nat Toxins. 1999; 8: 135–140. 10091133

[13] Rusmili MR, Yee TT, Mustafa MR, Othman I, Hodgson WC. In-vitro neurotoxicity of two Malaysian krait species (*Bungarus candidus* and *Bungarus fasciatus*) venoms: neutralization by monovalent and polyvalent antivenoms from Thailand. Toxins (Basel). 2014; 6 (3): 1036–1048. 10.3390/toxins6031036 24625762 PMC3968375

[14] Rusmili MR, Yee TT, Mustafa MR, Hodgson WC, Othman I. Isolation and characterization of a presynaptic neurotoxin, P-elapitoxin-Bf1a from Malaysian Bungarus fasciatus venom. Biochem Pharmacol. 2014; 91(3): 409–416. 10.1016/j.bcp.2014.07.001 25064255

[15] Rusmili MR, Tee TY, Mustafa MR, Othman I, Hodgson WC. Isolation and characterization of alpha-elapitoxin-Bf1b, a postsynaptic neurotoxin from Malaysian Bungarus fasciatus venom. Biochem Pharmacol. 2014; 88(2): 229–236. 10.1016/j.bcp.2014.01.004 24440452

[16] Rossetto O, Morbiato L, Caccin P, Rigoni M, Montecucco C. Presynaptic enzymatic neurotoxins. J Neurochem. 2006; 97(6): 1534–1545. 10.1111/j.1471-4159.2006.03965.x 16805767

[17] Rusmili MR, Yee TT, Mustafa MR, Hodgson WC, Othman I. Proteomic characterization and comparison of Malaysian *Bungarus candidus* and *Bungarus fasciatus* venoms. J Proteomics. 2014; 110: 129–144. 10.1016/j.jprot.2014.08.001 25154052

[18] Oh AMF, Tan CH, Tan KY, Quraishi NH, Tan NH. Venom proteome of *Bungarus sindanus* (Sind krait) from Pakistan and in vivo cross-neutralization of toxicity using an Indian polyvalent antivenom. J Proteomics. 2019; 193: 243–254. 10.1016/j.jprot.2018.10.016 30385415

[19] Oh AMF, Tan CH, Ariaranee GC, Quraishi N, Tan NH. Venomics of *Bungarus caeruleus* (Indian krait): Comparable venom profiles, variable immunoreactivities among specimens from Sri Lanka, India and Pakistan. J Proteomics. 2017; 164: 1–18. 10.1016/j.jprot.2017.04.018 28476572

[20] Tan KY, Tan CH, Sim SM, Fung SY, Tan NH. Geographical venom variations of the Southeast Asian monocled cobra (*Naja kaouthia*): venom-induced neuromuscular depression and antivenom neutralization. Comp Biochem Physiol C Toxicol Pharmacol. 2016; 185–186: 77–86. 10.1016/j.cbpc.2016.03.005 26972756

[21] Chanhome L, Khow O, Puempunpanich S, Sitprija V, Chaiyabutr N. Biological characteristics of the *Bungarus candidus* venom due to geographical variation. J Cell Anim Biol. 2009; 3: 93–100.

[22] Skejic J, Hodgson WC. Population divergence in venom bioactivities of elapid snake *Pseudonaja textilis*: role of procoagulant proteins in rapid rodent prey incapacitation. PLoS One. 2013; 8: e63988 10.1371/journal.pone.0063988 23691135 PMC3653870

[23] Fry BG, Wickramaratna JC, Jones A, Alewood PF, Hodgson WC. Species and regional variations in the effectiveness of antivenom against the in vitro neurotoxicity of death adder (*Acanthophis*) venoms. Toxicol Appl Pharmacol. 2001; 175: 140–148. 10.1006/taap.2001.9233 11543646

[24] Laemmli UK. Cleavage of structural proteins during the assembly of the head of bacteriophage T4. Nature. 1970; 227: 680–685. 10.1038/227680a0 5432063

[25] Schneider CA, Rasband WS, Eliceiri KW. NIH Image to ImageJ: 25 years of image analysis. Nat Methods. 2012; 9: 671–675. 10.1038/nmeth.2089 22930834 PMC5554542

[26] Adiwinata R, Nelwan EJ. Snakebite in Indonesia. Acta Med Indones. 2015; 47: 358–365. 26932707

[27] Warrell DA, Looareesuwan S, White NJ, Theakston R, Warrell M, Kosakarn W, et al Severe neurotoxic envenoming by the Malayan krait *Bungarus candidus* (Linnaeus): response to antivenom and anticholinesterase. Br Med J (Clin Res Ed). 1983; 286: 678–680. 10.1136/bmj.286.6366.678 6402200 PMC1547089

[28] Crachi MT, Hammer LW, Hodgson WC. A pharmacological examination of venom from the Papuan taipan (*Oxyuranus scutellatus canni*). Toxicon. 1999; 37: 1721–1734. 10.1016/s0041-0101(99)00114-2 10519650

[29] Barber CM, Isbister GK, Hodgson WC. Solving the 'Brown snake paradox': in vitro characterisation of Australasian snake presynaptic neurotoxin activity. Toxicol Lett. 2012 210(3): 318–323. 10.1016/j.toxlet.2012.02.001 22343038

[30] Gutierrez JM, Lomonte B. Phospholipases A_2_: unveiling the secrets of a functionally versatile group of snake venom toxins. Toxicon. 2013; 62: 27–39. 10.1016/j.toxicon.2012.09.006 23025922

[31] Rouault M, Rash LD, Escoubas P, Boilard E, Bollinger J, Lomonte B, et al Neurotoxicity and other pharmacological activities of the snake venom phospholipase A_2_ OS2: the N-terminal region is more important than enzymatic activity. Biochemistry. 2006; 45: 5800–5816. 10.1021/bi060217r 16669624 PMC2796912

[32] Kini RM. Excitement ahead: structure, function and mechanism of snake venom phospholipase A_2_ enzymes. Toxicon. 2003; 42: 827–840. 10.1016/j.toxicon.2003.11.002 15019485

[33] Chaisakul J, Parkington HC, Isbister GK, Konstantakopoulos N, Hodgson WC. Differential myotoxic and cytotoxic activities of pre-synaptic neurotoxins from Papuan taipan (*Oxyuranus scutellatus*) and Irian Jayan death adder (*Acanthophis rugosus*) venoms. Basic Clin Pharmacol Toxicol. 2013; 112(5): 325–334. 10.1111/bcpt.12048 23311944

[34] Tan CH, Liew JL, Tan KY, Tan NH. Assessing SABU (Serum Anti Bisa Ular), the sole Indonesian antivenom: A proteomic analysis and neutralization efficacy study. Sci Rep. 2016; 6: 37299 10.1038/srep37299 27869134 PMC5116744

[35] Chaisakul J, Alsolaiss J, Charoenpitakchai M, Wiwatwarayos K, Sookprasert N, Harrison RA, et al Evaluation of the geographical utility of Eastern Russell's viper (*Daboia siamensis*) antivenom from Thailand and an assessment of its protective effects against venom-induced nephrotoxicity. PLoS Negl Trop Dis. 2019; 13: e0007338 10.1371/journal.pntd.0007338 31644526 PMC6850557

[36] Prasarnpun S, Walsh J, Awad SS, Harris JB. Envenoming bites by kraits: the biological basis of treatment-resistant neuromuscular paralysis. Brain. 2005; 128: 2987–2996. 10.1093/brain/awh642 16195243

[37] Chippaux JP, Williams V, White J. Snake venom variability: methods of study, results and interpretation. Toxicon. 1991; 29(11): 1279–1303. 10.1016/0041-0101(91)90116-9 1814005

[38] Wickramaratna JC, Fry BG, Hodgson WC. Species-dependent variations in the in vitro myotoxicity of death adder (*Acanthophis*) venoms. Toxicol Sci. 2003; 74: 352–360. 10.1093/toxsci/kfg144 12773755

[39] Winter KL, Isbister GK, McGowan S, Konstantakopoulos N, Seymour JE, Hodgson WC. A pharmacological and biochemical examination of the geographical variation of *Chironex fleckeri* venom. Toxicol Lett. 2010; 192: 419–424. 10.1016/j.toxlet.2009.11.019 19945518

[40] Kunalan S, Othman I, Syed Hassan S, Hodgson WC. Proteomic Characterization of Two Medically Important Malaysian Snake Venoms, *Calloselasma rhodostoma* (Malayan Pit Viper) and *Ophiophagus hannah* (King Cobra). Toxins (Basel). 2018; 10(11). 10.3390/toxins10110434 30373186 PMC6266455

[41] Oh AMF, Tan CH, Tan KY, Quraishi NH, Tan NH. Venom proteome of *Bungarus sindanus* (Sind krait) from Pakistan and in vivo cross-neutralization of toxicity using an Indian polyvalent antivenom. J Proteomics. 2019; 193: 243–254. 10.1016/j.jprot.2018.10.016 30385415

[42] Patra A, Chanda A, Mukherjee AK. Quantitative proteomic analysis of venom from Southern India common krait (*Bungarus caeruleus*) and identification of poorly immunogenic toxins by immune-profiling against commercial antivenom. Expert Rev Proteomics. 2019; 16: 457–469. 10.1080/14789450.2019.1609945 31002271

[43] Chapeaurouge A, Silva A, Carvalho P, McCleary R, Modahl C, Perales J, et al Proteomic Deep Mining the Venom of the Red-Headed Krait, *Bungarus flaviceps*. Toxins. 2018; 10: 373 10.3390/toxins10090373 30217057 PMC6162843

[44] Wong KY, Tan CH, Tan KY, Quraishi NH, Tan NH. Elucidating the biogeographical variation of the venom of *Naja naja* (spectacled cobra) from Pakistan through a venom-decomplexing proteomic study. J Proteomics. 2018; 175: 156–173. 10.1016/j.jprot.2017.12.012 29278784

[45] Khow O, Chanhome L, Omori-Satoh T, Ogawa Y, Yanoshita R, Samejima Y, et al Isolation, toxicity and amino terminal sequences of three major neurotoxins in the venom of Malayan krait (*Bungarus candidus*) from Thailand. J Biochem. 2003; 134: 799–804. 10.1093/jb/mvg187 14769867

[46] Kessler P, Marchot P, Silva M, Servent D. The three‐finger toxin fold: a multifunctional structural scaffold able to modulate cholinergic functions. J Neurochem. 2017;142: 7–18. 10.1111/jnc.13975 28326549

[47] Kini RM, Doley R. Structure, function and evolution of three-finger toxins: mini proteins with multiple targets. Toxicon. 2010; 56: 855–867. 10.1016/j.toxicon.2010.07.010 20670641

[48] Kini RM. Molecular moulds with multiple missions: functional sites in three‐finger toxins. Clin Exp Pharmacol Physiol. 2002; 29: 815–822. 10.1046/j.1440-1681.2002.03725.x 12165048

[49] Kuhn P, Deacon AM, Comoso S, Rajaseger G, Kini RM, Uson I, et al The atomic resolution structure of bucandin, a novel toxin isolated from the Malayan krait, determined by direct methods. Acta Cryst. 2000; 56: 1401–1407. 10.1107/S0907444900011501 11053837

[50] Nirthanan S, Charpantier E, Gopalakrishnakone P, Gwee MC, Khoo HE, Cheah LS, et al Candoxin, a novel toxin from *Bungarus candidus*, is a reversible antagonist of muscle (alphabetagammadelta) but a poorly reversible antagonist of neuronal alpha 7 nicotinic acetylcholine receptors. J Biol Chem. 2002; 277: 17811–17820. 10.1074/jbc.M111152200 11884390

[51] Karsani SA, Othman I. Isolation, complete amino acid sequence and characterization of a previously unreported post-synaptic neurotoxin–AlphaN3, from the venom of *Bungarus candidus*. Biochem Biophys Res Commun. 2009;389: 343–348. 10.1016/j.bbrc.2009.08.145 19728988

[52] Shan LL, Gao JF, Zhang YX, Shen SS, He Y, Wang J, et al Proteomic characterization and comparison of venoms from two elapid snakes (*Bungarus multicinctus* and *Naja atra*) from China. J Proteomics. 2016; 138: 83–94. 10.1016/j.jprot.2016.02.028 26924299

[53] Utkin YN, Kuch U, Kasheverov IE, Lebedev DS, Cederlund E, Molles BE, et al Novel long- chain neurotoxins from *Bungarus candidus* distinguish the two binding sites in muscle-type nicotinic acetylcholine receptors. Biochem J. 2019; 476: 1285–1302. 10.1042/BCJ20180909 30944155

[54] Chang CC. Neurotoxins with phospholipase A_2_ activity in snake venoms. Proc Natl Sci Counc Repub China B. 1985; 9: 126–142. 2996044

[55] Tsai IH, Tsai HY, Saha A, Gomes A. Sequences, geographic variations and molecular phylogeny of venom phospholipases and threefinger toxins of eastern India *Bungarus fasciatus* and kinetic analyses of its Pro31 phospholipases A2. FEBS J. 2007;l 274: 512–525. 10.1111/j.1742-4658.2006.05598.x 17166178

[56] Slowinski JB. A phylogenetic analysis of *Bungarus* (Elapidae) based on morphological characters. J Herpetol. 1994; 440–446.

